# Re-Expression of ERα and AR in Receptor Negative Endocrine Cancers *via* GSK3 Inhibition

**DOI:** 10.3389/fonc.2022.824594

**Published:** 2022-03-24

**Authors:** Vikas Sharma, Jayadev Joshi, I-Ju Yeh, YongQiu Doughman, Daniel Blankenberg, David Wald, Monica M. Montano

**Affiliations:** ^1^Department of Pharmacology, Case Western Reserve University School of Medicine, Cleveland, OH, United States; ^2^Genomic Medicine Institute, Cleveland Clinic Lerner Research Institute, Cleveland, OH, United States; ^3^Department of Pathology, Case Western Reserve University School of Medicine, Cleveland, OH, United States

**Keywords:** androgen receptor, estrogen receptor, Dnmt1, GSK3, breast cancer, prostate cancer

## Abstract

DNA methylation, catalyzed by DNA methyltransferase (DNMT), is a well-characterized epigenetic modification in cancer cells. In particular, promoter hypermethylation of *AR* and *ESR1* results in loss of expression on Androgen Receptor (AR) and Estrogen Receptor (ER), respectively, and is associated with a hormone refractory state. We now report that Glycogen Synthase Kinase 3 (GSK3) phosphorylates DNMT1 at S714, which is localized to a 62 amino acid region referred to as auto-inhibitory linker, which functions to occlude the DNA from the active site of DNMT1 to prevent the methylation of unmethylated DNA. Molecular Dynamics simulation indicates that phosphorylation at S714 resulted in conformational rearrangement of the autoinhibitory domain that inactivated its ability to block the methylation of unmethylated DNA and resulted in enhanced DNA binding. Treatment with a novel and more selective inhibitor of GSK3 resulted in decreased methylation of the promoter region of genes encoding the Androgen Receptor (AR) and Estrogen Receptor alpha (ERa) and re-expression of the AR and ERa in AR negative prostate cancer and ER negative breast cancer cells, respectively. As a result, concurrent treatment with the GSK3 inhibitor resulted in responsiveness of AR negative prostate cancer and ER negative breast cancer cells to inhibitors of the AR or ER, respectively, in *in vitro* and *in vivo* experimental models.

## Introduction

DNA methylation is well-studied epigenetic modification that plays a key role in the regulation of gene expression (1). In mammals, DNA methylation primarily occurs within CpGs and is catalytically executed by a family of enzymes called DNA methyltransferases, DNMTs, which are classified as either maintenance or *de novo* methyltransferases (2) Maintenance methyltransferase DNMT1, maintains DNA methylation pattern during replication throughout the life of an organism, whereas *de novo* methyltransferases DNMT3a and DNMT3b establish methylation pattern during gametogenesis. With S-adenosylmethionine (SAM) acting as a methyl donor, DNMTs catalyze the addition of a methyl group to the fifth carbon of the cytosine residue ring of a CpG dinucleotide, generating 5-methylcytosine (3, 4).

DNA methylation has epigenetically been implicated at a global and local level in carcinogenesis. Globally, DNA methylation, along with genetic alterations and histone modifications, can result in dysregulation of gene expression profiles leading to initiation, promotion, and progression of cancer. Locally, DNA hypomethylation is proposed to cause activation of oncogenes and genetic instability. Conversely, tumor suppressor gene promoters are hypermethylated in several cancers resulting in silencing (5–7). The Androgen Receptor (AR) is a hormonal response gene that is hypermethylated in androgen-resistant prostate cancer (PCa) (8). About 30% of invasive breast cancer (BCa) are hormone independent because they lack Estrogen Receptor (ER) expression due to hypermethylation of ER promoter (9, 10).

The DNA methylation function of DNMT1 is reported to be modulated by post-translational modifications, which include phosphorylation, ubiquitination, methylation and acetylation (2). Mass spectrometry analyses indicate that human DNMT1 activity is regulated at the post-translational level through phosphorylation at the N-terminal domain (2), and phosphorylation by kinases Akt and PKC at this domain resulted in disruption of DNMT1 interaction with its partners and promoted global hypomethylation (11). Phosphorylation of DNMT1 at the C-terminal domain influenced the abundance and activity of DNMT1 (2).

Glycogen Synthase Kinase 3 (GSK3) regulates a large number of cellular processes and has two isoforms: GSK3α and GSK3β. A study reported that GSK3β has more predicted substrates than any other kinase (12). GSK3 is constitutively active in the cell, and it is inhibited, rather than activated, in response to stimulation by growth factors and the Wnt pathway (13). GSK3 has been mainly considered a cytosolic protein, but it is also localized within the mitochondria and nucleus. When localized in the nucleus, GSK3 regulates several transcription factors, including DNA-modifying enzymes. In particular, GSK3 is reported to phosphorylate and/or reduce the stability of DNMT1 (14, 15). GSK3 also regulates the stability of DNMT3a2, although the mechanism is unknown (16). Thus, by regulating transcription factors and proteins involved in epigenetic regulation, GSK3 has diverse modulatory effects on gene expression.

We now report that GSK3 phosphorylates DNMT1 at S714, which is localized to a 62 amino acid region referred to as auto-inhibitory linker, which functions to occlude the DNA from the active site of DNMT1 to prevent the methylation of unmethylated DNA (17, 18). Our Molecular Dynamics (MD) simulation indicates that phosphorylation at S714 resulted in conformational rearrangement of the autoinhibitory domain, attenuated its ability to block the methylation of unmethylated DNA, and resulted in enhanced DNA binding. Inhibition of GSK3 and the ensuing inhibition of DNMT1 activity resulted in re-expression of the Androgen Receptor (AR) and Estrogen Receptor (ER) in prostate cancer (PCa) and breast cancer (BCa) cells, respectively.

## Materials and Methods

### Cell Culture and Treatments

PCa and Triple Negative Breast Cancer (TNBC) cell lines were purchased from the American Type Culture Collection (ATCC, Manassa, VA, USA) and were maintained as recommended by ATCC and authenticated using STRS analyses. Cells were utilized within 10 passages of the stock obtained from ATCC. Cell lines were tested negative for Mycoplasma using MycoAlert Mycoplasma Detection Kit (Lonza).

Kinase inhibitors and their sources are: CHIR-99021 (Tocris Bioscience, Minneapolis, MN, USA, catalog #4423) and RO-3306 (MedChem Express, Monmouth Junction, NJ, USA, catalog # HY-12529). 5-Aza-2’deoxycytidine (DNMT1 inhibitor) was obtained from MP Biomedicals (Solon, OH, USA). GSK3 inhibitor 71 is a proprietary compound obtained from CuronBiotech (Cleveland, OH, USA) and is a highly potent and specific for GSK3. GSK3i 71 demonstrated inhibition of GSK3α and GSK3β (IC50 146 pM and 144 pM respectively). Importantly when kinase profiling was performed (on a panel of 100 kinases at 1 µM), besides GSK3α and GSK3β only a single kinase (Cyclin Dependent Kinase 1, CDK16) exhibited inhibition >50%. Follow-up IC50 testing on CDK16 testing revealed >2700-fold specificity of GSK3i 71 to GSK3 (IC50 of 401 nM for CDK16). Of note, GSK3i 71 did not lead to significant inhibition of any other CDK’s tested (CDK2, 3, 5, 7, 9, 11). Further illustrating the high level of specificity of GSK3i 71, a recent report describing a GSK3 inhibitor with “exquisite selectivity” reports a fold selectivity of GSK3 *vs* CDK’s of 41-fold (19).

### Site-Directed Mutagenesis

Myc-DNMT1 was purchased from Addgene (plasmid #36939). Myc-DNMT1 (S714A) and Myc-DNMT1 (S714D) were generated using wild-type Myc-DNMT1 as a template and the QuikChange II XL Site-Directed Mutagenesis Kit (Agilent Technologies, Santa Clara, CA, USA, catalog #200521), according to the manufacturer’s protocol. Introduction of the desired mutation and lack of secondary mutations were verified by DNA sequencing. Cells were transfected with expression vector for Myc-DNMT1, Myc-DNMT1 (S714A) or Myc-DNMT1 (S714D) using Lipofectamine 2000 (Thermo Scientific, Waltham, MA, USA, catalog #L3000015).

### Lentiviral Infection

Plasmids used to generate GSK3α and GSK3β shRNA lentiviruses were obtained from Sigma-Aldrich (St. Louis, MO, USA). Lentiviral particles were generated by transfecting HEK293FT cells with GSK3α or GSK3β shRNA expression plasmid along with two other plasmids, envelope expressing plasmid (pMD2.G), and packaging plasmid (psPAX2), using Lipofectamine 3000. Following forty to sixty hours of transfection, medium containing lentivirus was collected. C4-2, DU145, and MDA-MB-231 cells were transduced with lentiviral particles in the presence of polybrene (10 µg/ml) for 18–24 h. Transduced cells were selected using puromycin, harvested, and processed for immunoblotting to confirm knockdown of targeted proteins.

### Co-Immunoprecipitation

Endogenous proteins were co-immunoprecipitated and analyzed as described previously (20).

### MD (Molecular Dynamics) Simulation

Crystal structure of DNMT1 protein complex with DNA (PDB ID: 3PTA) resolved at 3.60 Å was obtained from PDB (17) (21). Inputs for MD simulation from phosphorylated and unphosphorylated DNMT1 protein were prepared using Charmm-GUI (https://www.charmm-gui.org/) before MD simulation (22). Protein-DNA complex was dissolved at the center of a cubic box and solvated with single point charge water molecules with a 1.0 nm solute-box distance. Simulation studies were carried out on GROMACS 2020.6 using charmm-36 force field (23). Periodic boundary conditions were applied to simulate the protein-DNA complex in order to deal with surface effects. Counter ions were added to neutralize the effect of charge on the protein-DNA complex before a short energy minimization, 50000 steps, using the steepest descent algorithm. Parameters for NVT (constant Number of particles, Volume, and Temperature), NPT (constant Number of particles, Pressure, and Temperature) and production run were adopted (24). During the production run, simulated coordinates of the system were captured at every 0.1 ns. Finally, to analyze the simulated system, we used the built-in functionality of the GROMACS package and MDAnalysis package, a python library (25). MD simulations were conducted on a Dell workstation with MPI, and GPU enabled mode. We achieved 21.47 ns/day maximum performance speed during MD simulation.

### Expression and Purification of GST-Tagged Constructs

The GST-DNMT1 (431-836) plasmid was generously provided by Dr Pierre Esteve (New England Biolabs, Ispwich, MA, USA). GST-tagged DNMT1 constructs were expressed in E. coli BL21 cells. Recombinant proteins were purified using Glutathione Sepharose beads (Sigma Aldrich catalog #GE17-0756-01) and detected by western blotting using GST monoclonal antibody (Cell Signaling Technology, Danvers, MA, USA, catalog #2624).

### *In Vitro* Kinase Assay

Recombinant active GSK3β protein (0.1 µg) was incubated with 1 µg of purified GST-tagged DNMT1 proteins in the presence of 100 µM ATP in kinase buffer (50 mM β-glycerophosphate, pH 7.4; 10 mM MgCl2; 10 mM NaF; 1 mM DTT). Reactions were incubated at 30°C for 30 minutes and terminated by addition of equal volume of 10 mM EDTA. Reaction products were analyzed using an ELISA essay wherein 96-well plates were coated with 100 ng/well of GST monoclonal antibody at 4°C overnight and incubated with blocking buffer (2% BSA, 0.1% Tween 20) for 1 h. Diluted kinase products were loaded onto the coated wells and incubated for 2 hours at room temperature, followed by the addition of phosphoserine antibody (catalog number), and incubation for an additional 2 hours at room temperature. HRP-conjugated secondary antibody in blocking buffer was added and incubated for 1 h at room temperature. Reaction products were detected by the addition of 100 µl chromogenic substrate (3,3’,5,5’- tetramethylbenzidine), TMB for 30 min. The reaction was terminated with 50µl of 1 M HCl and absorbance at 450 nm was measured using a Molecular Devices plate reader (San Jose, CA, USA).

### qRT- PCR

Total RNA was extracted using Trizol (Invitrogen) according to the manufacturer’s instructions, and 2 µg of RNA was reverse-transcribed into complementary DNA, which was amplified using qRT-PCR. The mRNA expression levels of *ESR1* or *AR* gene were normalized to the expression level of the housekeeping gene *GAPDH* using the following primers:

hESR1 fw: ATGTGCCTGGCTAGAGATCC
hESR1 rv: CAAACTCCTCTCCCTGCAGA
hAR fw: TGCTCCGCTGACCTTAAAGA
hAR rv: GCCTTACACAACTCCTTGGC
hGAPDH fw: GTCATCATCTCTGCCCCCTCTGCT
hGAPDH rv: CTTCTTGATGTCATCATATTTG

### Bisulfite Modification and Methylation-Specific PCR

Total genomic DNA was extracted from cells by using the Gentra Puregene kit (Qiagen) following the manufacturer’s recommendations. DNA was subjected to bisulfite conversion using EZ DNA Methylation kit (ZYMO Research, Orange, CA) according to the manufacture’s instruction. For PCR amplification of the methylated and unmethylated ESR1 promoter, RT-PCR was performed on the bisulfite converted DNA with the following primers:

methylated ESR1 fw: CGAGTTGGAGTTTTTGAATCGTTC
methylated ESR1 rv: CTACGCGTTAACGACGACCG
unmethylated ESR1 fw: ATGAGTTGGAGTTTTTGAATTGTTT
unmethylated ESR1 rv: ATAAACCTACACATTAACAACAACCA
methylated AR fw: CGTTTTTTTCGAGATTTCG
methylated AR rv: CGAACGACGACTTCGAAACCG
unmethylated AR fw: TGTTTTTTTTGAGATTTTG
unmethylated AR rv: CAAACAACAACTTCAAAACCA

PCR products were resolved on a 2.5% agarose gel by gel electrophoresis.

### Western Blotting Analyses

Whole cell lysates were prepared from cells and subjected to western blot analyses as previously described (26). Western blots were incubated with primary antibodies from Cell Signaling Technology [anti-CDK1 (catalog #77055), anti-ERα (anti-AR, anti-PSA (catalog #5365), and anti-c-Myc (catalog #9402)], from Santa Cruz Biotechnology (Dallas, TX, USA) [anti-GSK3 (catalog #sc-7291), anti-AR (catalog #sc-7305), and anti-ERα (catalog #sc-543)], from Invitrogen [anti-DNMT1 (catalog #PA5-80557)], from Millipore [phosphoserine(catalog #AB1603)] and from Sigma-Aldrich [anti-GAPDH (catalog #MAB374)]. Anti-pDNMT1 (S714) is an in-house antibody. Blots were then incubated with anti-rabbit or anti-mouse secondary antibody (catalog #31460 and 31430, respectively, Thermo Scientific, Waltham, MA, USA) at room temperature. Signals were detected using SuperSignal™ West Femto Maximum Sensitivity Substrate (Thermo Scientific, catalog #34094), visualized using the LI-COR Odyssey System (LI-COR Biosciences, Lincoln, NE, USA), and quantified using the ImageJ software.

### DNMT1 Activity

C4-2 and MDA-MB-231 cells were transfected with Myc-DNMT1-WT, Myc-DNMT1-S714A, Myc-DNMT1-S714D, and/or GSK3 shRNA. Following transfection, cells were harvested, and nuclear extracts were prepared using nuclear extraction kit (Millipore, Burlington, MA, USA, catalog #2900) as per the manufacturer’s protocol. DNA methyltransferase activity was measured by a colorimetric DNMT activity assay (Active Motif, Carlsbad, CA, USA, catalog #55006) wherein the methylation of CpG-rich sequences that were immobilized on 96-well plates was assessed. DNMT1 transfers the methyl group from S-Adenosyl methionine (SAM) to the immobilized DNA, and the methylated cytosine was quantified by ELISA with a specific methyl-binding protein and HRP-conjugated antibody.

### Chromatin Immunoprecipitation (ChIP) Analysis

DU145 and MDA-MB-231 cells were grown in 15 cm^2^ dishes and transfected with Myc-DNMT1 (WT), Myc-DNMT1 (S714), Myc-DNMT1 (S714D), or transduced with lentiviruses expressing control shRNA or GSK3 shRNA. Thereafter, cells were processed for ChIP analyses as described previously (27) to assess recruitment of Myc-DNMT1 and DNMT1 to the promoter regions of *AR* and *ESR1* that have been reported to be recruitment sites for DNMT1 (28–30). Antibodies utilized were from Santa Cruz Biotechnology: normal mouse IgG (catalog #sc-2025), anti-c-Myc (catalog #sc-40), and anti-DNMT1 (catalog #sc-271729). The following primers were used to amplify the indicated gene regions:

*AR* promoter:
5′-CGACTCGCAAACTGTTGCATT-3′
5′-AAAGGCAGCCGTCAGTCCTA-3′
*ESR1* promoter:
5′-GAACCGTCCGCAGCTCAAGATC-3′
5′-GTCTGACCGTAGACCTGCGCGTTG-3′

### Cellular Proliferation Using WST-1 Reagent

Cells were plated onto a 96-well plate at a density of 3000 cells/well. Some cells were infected with control shRNA or GSK3 shRNA lentiviruses. Transduced cells were selected using puromycin prior to plating onto 96-well plates for proliferation assays. Media containing DMSO, R1881, or Bicalutamide were replaced every other day. After 6 days, WST-1 solution (Sigma Aldrich, catalog # 11644807001) was added to each well. Absorbance was recorded at 450 nm wavelength using a Molecular Devices plate reader (San Jose, CA, USA).

### Colony Formation Assay

Five hundred cells are aliquoted onto 12-well plates and fed fresh growth media containing GSK3i 71 in the absence or presence of antagonists for the ER or AR every 3–4 days. After 2 weeks of culture, cells were fixed with methanol in room temperature for 20 minutes, stained with methylene blue, and colonies were photographed.

### Xenograft Studies

All animal procedures were approved by the CWRU Institutional Animal Care and Use Committee. MDA-MB-231 cells (1x10^6^) in 100 ul PBS: Matrigel (growth factor-reduced; Corning, Corning, NY, USA, catalog #356231) were injected subcutaneously into the flank of 8-week old female NOD scid gamma (NSG) mice. When tumors reached a size of approximately 100 mm^3^, mice were randomized and matched into treatment groups (5-6 mice/treatment group). Tamoxifen (75 mg/Kg), GSK3i 71 (10 mg/Kg), or a combination of both were administered by intraperitoneal injection daily for 4 weeks. DU145 cells (1x10^6^) in 100 ul PBS: Matrigel were injected subcutaneously into the flank of 8-week old male NSG mice. When tumors reached a size of approximately 100 mm^3^, mice were randomized and matched into treatment groups (5-6 mice/treatment group). Enzalutamide (30 mg/Kg), GSK3i 71 (10 mg/Kg), or a combination of both were administered by intraperitoneal injection daily for 4 weeks.

Tumors were measured weekly by caliper. Tumor volumes were calculated by the following formula: a^2 × b × 0.5, where a is the smallest diameter and b is the diameter perpendicular to a. Body weights were taken weekly and no significant differences in body weight were noted between groups. Tumors were collected and weighed at study termination, processed for immunohistochemistry, or snap-frozen in liquid nitrogen and stored at 80 C for Western blot analyses.

### Immunohistochemistry

MDA-MB-231 and DU145 xenografts were fixed in 10% formalin and embedded with paraffin. Embedded tissues were sectioned (10 µm) for immunohistochemistry experiments. Prior to immunostaining, paraffin sections were deparaffinized and rehydrated. Paraffin sections were first boiled in 10 mM citrate buffer (pH 6.0) to retrieve antigenicity. Sections were incubated in 3% H_2_O_2_ in PBS for 10 min at room temperature to quench endogenous peroxidase and incubated in 10% normal serum (prepared from the species in which the secondary antibodies were generated) for 1 h at room temperature to block the non-specific binding sites. Sections were incubated with the primary antibody for 1 hr at room temperature or overnight at 4 C. Antibodies were obtained from Santa Cruz Biotechnology (Santa Cruz, CA): anti-AR (catalog #sc-7305), and anti-ERα (catalog #sc-543). Following washes, sections were treated with secondary antibody for 1 h at room temperature. Then the Vectastain ABC kit (Vector Laboratories, Burlingame, CA) was used per manufacturers’ instructions. Peroxidase activity was visualized with 3,3’-diaminobenzidine. Negative controls were incubated with non-specific IgG.

### Statistical Analysis

All experiments have been performed at least three times independently and are represented as Mean ± SE. Statistical significance was determined using Student’s *t* test comparison. Significance of differences was determined by one-way ANOVA followed by multiple comparisons. P< 0.05 was accepted as an appropriate level of significance.

## Results

### Identification of DNMT1 as a Target of GSK3

Due to our interest in differentiating agents, we used a phosphoproteomic approach to determine kinases that are potentially targeted by the differentiating agent hexamethylene bisacetamide (HMBA) in TNBC cells, MDA-MB-231. Mass spectrometry studies revealed that peptide fragments of DNMT1 phosphorylated at S714 were enriched in vehicle treated cells when compared to HMBA-treated cells. Regulation of the phosphorylation of DNMT1 at S714 by RAS and SET were reported in a recent publication and in a preprint (31, 32). Using HPRD v.9, we identified other possible kinases that can phosphorylate DNMT1 at S714, and among the highest scoring kinases were GSK3 and Cyclin Dependent Kinase 1 (CDK1).

**Figure 1:**
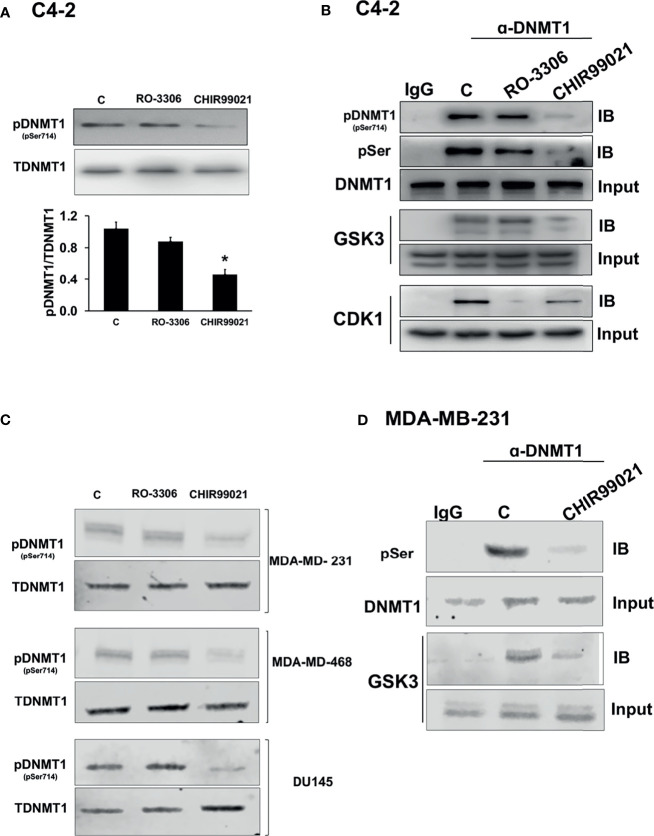
Regulation of the levels of phosphorylated DNMT1 (S714). **(A)** Immunoblot analysis of phosphorylated DNMT1 (S714) in C4-2 cells treated with either CHIR99021 (4 µM) or RO-3306 (1 µM) for 4 h *P < 0.05 *vs*. Control. Figures are representative of at least 3 independent experiments. **(B)** C4-2 cells were treated with either RO-3306 (4 µM) or CHIR-99021 (4 µM) for 4 h to inhibit CDK1 and GSK3, respectively. Cell extracts were immunoprecipitated with DNMT1 prior to immunoblot analyses of pDNMT1 (S714), phosphorylated serine (pSer), DNMT1, GSK3, and CDK1. **(C)** Immunoblot analysis of phosphorylated DNMT1 (S714) in MDA-MB-231, MDA-MB-468, and DU145 cells treated with either CHIR99021 (4 µM) or RO-3306 (1 µM) for 4 h **(D)** MDA-MB-231 cells were treated with CHIR-99021 (4 µM) for 4 h to inhibit GSK3. Cell extracts were immunoprecipitated with DNMT1 prior to immunoblot analyses of phosphorylated serine (pSer), DNMT1, GSK3, and CDK1. Figures are representative of at least 3 independent experiments.

To test which kinase phosphorylated DNMT1 at S714 we treated PCa C4-2 cells with either a CDK1 inhibitor, RO-3306, or a GSK3 inhibitor, CHIR99021 and assessed the levels of phospho-DNMT1 (S714). While CDK1 inhibition had no effect on levels of phospho-DNMT1 (S714), GSK3 inhibition significantly reduced the levels of phospho-DNMT1 (S714), detected using anti-pDNMT1 S714 or anti-phosphoserine (**Figure 1**). Moreover, co-immunoprecipitation experiments indicate that GSK3 inhibition resulted in attenuation of the interaction of GSK3 with DNMT1, accompanied by a significant reduction in phosphorylation of DNMT1 at S714 (**Figure 1B**). These results provide support for the phosphorylation of DNMT1 at S714 by GSK3, and phosphorylated DNMT1 more strongly interacts with GSK3. Another implication from these findings is a positive feedback loop that results in enhanced phosphorylation of DNMT1. We also detected an interaction between DNMT1 and CDK1, and the interaction was attenuated in cells treated with CDK1 inhibitor, but no decrease in expression of phospho-DNMT1 (S714) was evident (**Figure 1B**). Reduction in the levels of phospho-DNMT1 (S714) upon GSK3 inhibition was also evident in TNBC cells, MDA-MB-231 and MDA-MB-468, and AR negative DU145 PCa cells (**Figure 1C**). As what was observed in C4-2 cells, GSK3 inhibition resulted in attenuation of the interaction of GSK3 with DNMT1 in MDA-MB-231 cells (**Figure 1D**).

**Figure 2:**
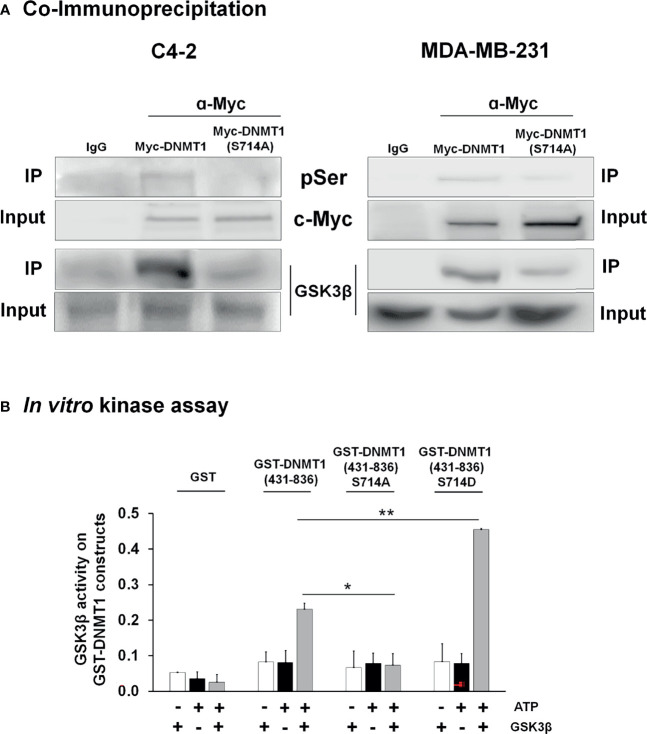
GSK3 interacted with and phosphorylated DNMT1 **(A)** C4-2 and MDA-MB-231 cells were transfected with expression vector for either Myc-DNMT1 (WT) or Myc-DNMT1 (S714A). Myc-tagged proteins were immunoprecipitated using c-Myc antibody. Shown are immunoblot analyses of phosphorylated serine (pSer), Myc-tagged DNMT1, and GSK3β. Figures are representative of at least 3 independent experiments. **(B)** *In vitro* kinase assays were performed using recombinant GSK3β (0.1 µg), 100 µM ATP, and purified GST-DNMT1 constructs (1 µg) for 30 min at 30°C. Phosphoserine signal in reaction products was detected by ELISA wherein wells were coated with anti-GST antibody and reaction products incubated with anti-phosphoserine and HRP-linked secondary antibody. Negative controls in each experiment are samples lacking ATP or recombinant GSK3β. *P < 0.05 and **P < 0.01 *vs*. GST-DNMT1 (WT). Figures are representative of at least 3 independent experiments.

To verify phosphorylation of DNMT1 at S714 by GSK3 we generated a DNMT1 point mutant by replacing S714 with an alanine and introduced expression plasmids for Myc tagged-DNMT1-WT or DNMT-S714A into C4-2 and MDA-MB-231 cells. Myc-tagged DNMT1 proteins were immunoprecipitated using Myc antibody. pSer antibody was used to detect phospho-DNMT1 and GSK3β antibody was used to detect the interaction of DNMT1 with GSK3β. Our results in both cell lines are consistent and showed significant reduction in the levels of serine phosphorylated DNMT1 in DNMT1-S714A expressing cells and that S714A mutation resulted in attenuated interaction of GSK3β with DNMT1 (**Figure 2**).

An *in vitro* kinase assay was used to demonstrate the ability of GSK3 to phosphorylate S714 of DNMT1. We also generated a phosphomimetic mutant, GST- DNMT1-S714D, to test in this assay. Recombinant GSK3β was incubated with purified GST- DNMT1 (431-836)- WT, - S714A, or -S714D. As shown in **Figure 2B**, GSK3β catalyzed the phosphorylation of DNMT1 WT but not DNMT1 S714A. The phosphorylation of DNMT1 S714D was augmented when compared to DNMT1 WT, perhaps due to enhanced interaction between S714D and GSK3 and subsequent phosphorylation of DNMT1 at other sites. This is consistent with the attenuation of the interaction of GSK3 with DNMT1 upon GSK3 inhibition or S714A mutation (**Figure 1B**).

### GSK3-Induced Phosphorylation of DNMT1 Resulted in Enhanced Binding to Promoter Regions *AR* and *ESR1*

**Figure 3:**
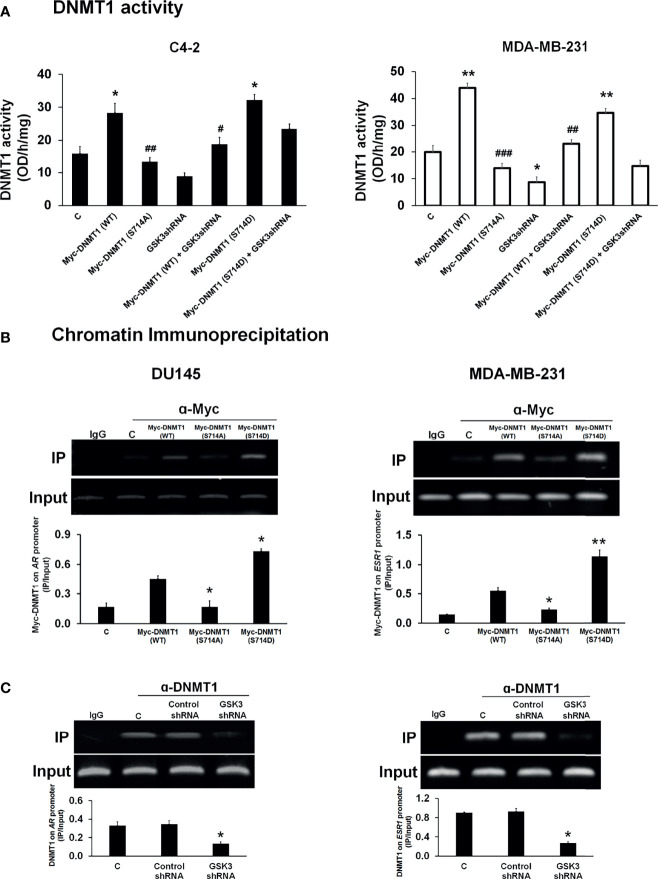
Modulation of DNMT1 activity and DNA binding by phosphorylation at S714. **(A)** Nuclear extracts were generated from C4-2 and MDA-MB-231 cells transfected with expression vectors for Myc-DNMT1 (WT), Myc-DNMT1 (S714A), Myc-DNMT1(S714D) and/or transduced with GSK3 shRNA lentiviruses. DNMT activity (methylation of CpG-rich sequences immobilized on 96-well plates) was assessed according to the manufacturer’s protocol. Figures are representative of at least 3 independent experiments. *P < 0.05 and **P < 0.01 *vs*. Control. ^#^P < 0.05 and ^##^P < 0.01, and ^###^P < 0.001 *vs*. DNMT1 (WT). **(B)** DU145 and MDA-MB-231 cells were transfected with expression vectors for Myc-DNMT1 (WT), Myc-DNMT1 (S714A), or Myc-DNMT1(S714D). Cells were processed for ChIP analyses of the recruitment of Myc-tagged proteins on the *AR* promoter in DU145 cells and on the first exon of the *ESR1* gene in MDA-MB-231 cells. Input DNA was used as normalization control. IgG control was used as a negative control. The gel images are representatives of 3 independent experiments. *P < 0.05 and **P < 0.01 *vs*. Myc-DNMT1 (WT). **(C)** DU145 and MDA-MB-231 cells were infected with control or GSK3 shRNA lentiviruses targeting GSK3α and GSK3β and selected using puromycin. Cells were then processed for ChIP analyses of recruitment of DNMT1 to the *AR* promoter in DU145 cells and to the first exon of the *ESR1* gene in MDA-MB-231 cells. Input DNA was used as normalization control. IgG control was used as a negative control. Figures are representative of at least 3 independent experiments. *P < 0.05 *vs*. Control.

We then determined the effect of phosphorylation at S714 on DNMT1 activity in C4-2 and MDA-MB-231 cells. To do so, we generated a phosphomimetic mutant [Myc-DNMT1 (S714D)]. We observed that nuclear extracts generated from cells transfected with either WT-DNMT1 or DNMT1 (S714D) exhibited increased DNMT1 activity when compared to extracts from cell transfected with DNMT1 (S714A) (**Figure 3A**). Altered DNMT1 activity cannot be attributed to differences in expression levels of WT and mutant DNMT1 (**Supplementary Figure 1A**). Cells expressing GSK3 shRNAs targeting GSK3α and GSK3β (**Supplementary Figure 1B**) exhibited attenuated DNMT1 activity (**Figure 3A**). Co-expression with DNMT1 (S714D) rescued the decrease in DNMT1 activity caused by targeting GSK3, more so in C4-2 cells than in MDA-MB-231 cells (**Figure 3A**).

Because of the important role of the Estrogen Receptor and Androgen Receptor in hormone dependent cancers, and the reported loss of expression of these receptors due to DNMT1-mediated promoter hypermethylation we examined if phosphorylation of DNMT1 will alter its ability to silence the expression of these hormone receptors. We expressed DNMT1 WT and DNMT1 mutants (S714A and S714D) in DU145 and MDA-MB-231 cells (**Supplementary Figure 2A**) and examined binding to CpG rich regions reported to be sites of DNMT1 binding on the *AR* and *ESR1* promoters (28–30). Recruitment of DNMT1 (WT) to the *AR* and *ESR1* promoters was observed in DU145 and MDA-MB-231 cells (**Figure 3B**). The phosphomimetic mutant DNMT1 S714D exhibited enhanced binding when compared to WT DNMT1, while DNMT1 S714A exhibited weaker binding to *AR* and *ESR1* promoters (**Figure 3B**). The critical role of GSK3-mediated DNMT1 S714 phosphorylation and the ensuing enhanced binding to DNA was supported by attenuated binding of DNMT1 to *AR* and *ESR1* promoters upon knockdown of GSK3 in DU145 and MDA-MB-231 cells (**Figure 3C** and **Supplementary Figure 2B**). GSK3 inhibition, but not CDK1 inhibition, also resulted in attenuated binding of DNMT1 to *AR* and *ESR1* promoters (**Supplementary Figure 2C**). 

### Stabilization of the Autoinhibitory Domain by Phosphorylation

**Figure 4:**
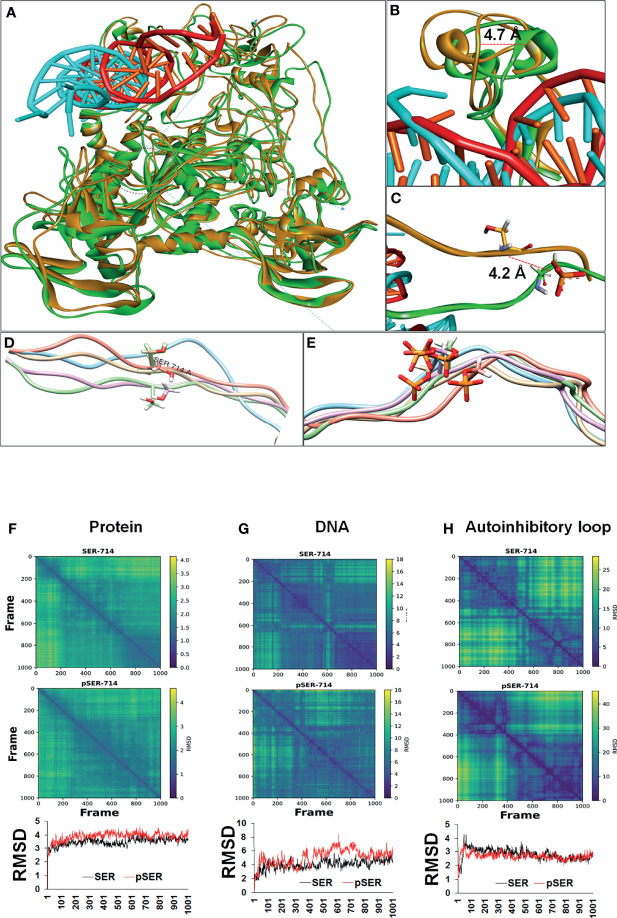
Conformational changes resulting from phosphorylation of DNMT1 based on MD simulation. **(A)** Superimposed conformation of unphosphorylated (orange) and phosphorylated (green) protein-DNA complex at 80 ns. DNA (Cyan) bound with phosphorylated protein shows significant conformational changes. **(B)** The CXXC region (green) which holds DNA shows 4.7Å displacement in the phosphorylated protein. **(C)** After phosphorylation, the flexible autoinhibitory domain was displaced by 4.2 **(A, D, E)** A zoomed image of the flexible loop (20 AA) spanning phosphorylated residues shows stable conformation in the case of phosphorylated residue represented in stick model with orange color. **(F-H)** Diffusion map based on distance matrix and RMSD analysis. **(F)** The column represents global changes in the protein chain. No huge differences were observed, however, a relatively stable global conformation of phosphorylated protein was observed in comparison to unphosphorylated protein. **(G)** DNA bound with phosphorylated residue depicts large conformational changes followed by a stable state. **(H)** The local effect of phosphorylation shows a relatively stable conformation of the autoinhibitory loop (20 AA), which is involved in autoinhibition.

To gain insight into the structural basis for enhanced DNMT1 binding to DNA upon phosphorylation, MD simulation study was done to capture the conformational changes after the phosphorylation (S714 to pS714) of the DNMT1 protein-DNA complex. Trajectory file was obtained by saving the coordinates at every 0.1 ns interval during simulation. Superimposed simulated structures at 80 ns are represented in **Figure 4A**. Local and specific changes around the phosphorylated residues were visually analyzed by superimposing the simulated coordinates of both the complexes captured at every 20 ns interval during the 100 ns MD simulation, and results indicated that phosphorylation at S714 stabilizes the flexible loop of the autoinhibitory domain after an initial structural rearrangement (**Figures 4D, E**). The overall impact of phosphorylation on the 100-amino acid region, which included the flexible autoinhibitory loop and the CXXC domain, is visually represented in **Supplementary Figures 3A, C, D**). However, at the global level, the protein backbone remains stable (**Figure 4A**). As compared to the overall protein structure, DNA moiety (cyan color) of the protein-DNA complex depicts relatively higher conformational changes (**Figure 4A** and **Supplementary Figure 3B**).

After MD simulation, effect of phosphorylation on the overall conformation of the protein-DNA complex was assessed based on diffusion map and root-mean-square deviation (RMSD). Similar to the visual analysis, diffusion map and RMSD analysis of the protein-DNA complex indicated that the phosphorylation stabilized the protein conformation (**Figure 4F**). However, these stabilization effects are less visible at the entire protein but relatively high at the local level (**Figure 4H**). Local changes were assessed based on 20 amino acids, spanning the phosphorylated residue equally towards C and N terminal (**Figures 4D, E, H**). These local changes led to the conformational rearrangement of the autoinhibitory domain (**Figure 4B**).

Unlike the protein, DNA (cyan) shows relatively wide conformational changes during the simulation of phosphorylated protein (30-80 ns) (**Figures 4A, G**). DNA-bound unphosphorylated protein (red) does not show noticeable conformational changes and remains rather stable (**Figures 4A, B**.) Conversely, DNA bound with phosphorylated protein shows a large conformational shift (40 ns-75 ns) and achieves a stable state eventually (75-100 ns) (**Figures 4A, G**). Overall, MD simulation analysis indicated that the phosphorylation leads to stabilization of the autoinhibitory linker (loop) and the CXXC domain after a conformational rearrangement that leads to a 4.2 Å and 4.7 Å displacement of these two regions respectively (**Figures 4B, C**). These conformational changes within the protein resulted in conformational rearrangement of the DNA moiety of the complex, which may result in higher activity of the DNMT1 protein.

### Restoration of AR Expression and Response to an Anti-Androgen in AR-Negative Cell Line

Initially, PCa are sensitive to androgens, resulting in hormone-induced proliferation that can be inhibited by androgen deprivation therapy (33). However, as the disease progresses, therapeutic inhibition of AR signaling results in PCa that de-differentiate into highly aggressive AR-negative disease (33). One of the factors that contribute to this transition is aberrant DNA methylation of the AR gene promoter that results in the loss of the *AR* gene expression in PCa (34). Thus, the re-expression of AR *via* DNMT1 inhibitors has been evaluated as a therapeutic goal in clinical trials.

**Figure 5:**
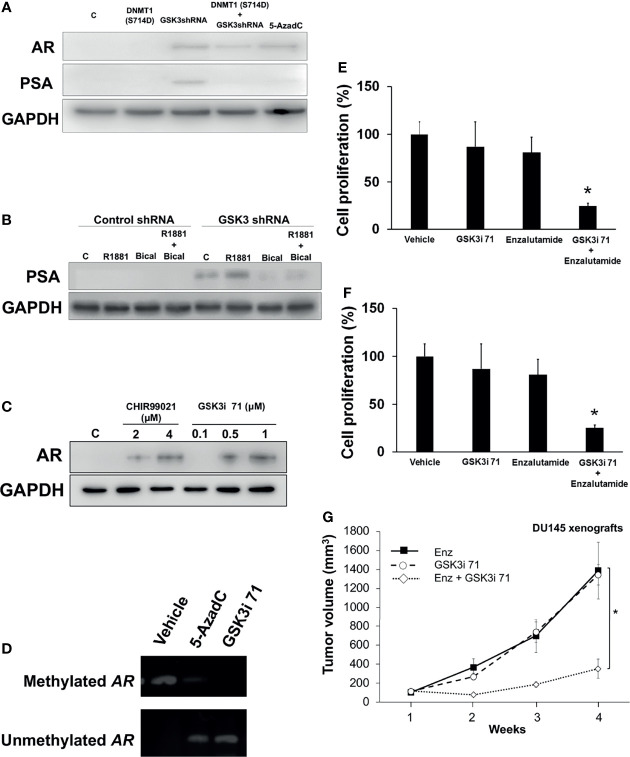
GSK3 inhibition resulted in re-expression of AR and hormone dependence of an AR negative cell line. **(A)** DU145 cells were transfected with expression vector for DNMT1 (S714D) and/or GSK3 shRNAs targeting GSK3α and GSK3β and processed for western blot analyses of AR and PSA expression normalized to GADPH. Cells treated with 5-AzadC (5 µM for 48 h) were used as positive controls. Figures are representative of at least 3 independent experiments. **(B)** DU145 cells were transduced with control or GSK3 shRNA lentiviruses targeting GSK3α and GSK3β followed by puromycin selection. Some cells were treated with R1881 (5 nM, 4 h) and/or Bicalutamide (5 µM, 4 h). Cell were processed for western blot analyses of PSA expression normalized to GAPDH. Figures are representative of at least 3 independent experiments. **(C)** Cells were treated with indicated concentrations of GSK3 inhibitors. Cell were processed for western blot analyses of AR expression normalized to GAPDH. **(D)** DU145 cells were treated with vehicle, 5-AzadC (5 µM), or GSK3i 71 (500 nM) for 72 h Cells were harvested and genomic DNA extracted, followed by methylation-specific PCR at the promoter region of the *AR* gene using bisulfite-treated DNA. Figures are representative of at least 3 independent experiments. **(E)** DU145 cells were infected with control or GSK3 shRNA-containing lentiviruses targeting GSK3α and GSK3β followed by selection using puromycin and plating for cell proliferation. Some cells were treated with R1881 or Bicalutamide. **(F)** DU145 cells were treated with Enzalutamide (10 µM) and/or GSK3i 71 (500 nM). For **(E, F)**, after 5 days of treatment, WST-1 solution (10 mg/ml) was added to each well. The formazan crystals formed inside the viable cells were measured at 450 nm using a microplate reader. Figures are representative of at least 3 independent experiments *P < 0.05 *vs*. GSK3 shRNA. **(G)** Male NSG mice were implanted with DU145 cells and treated Enzalutmide (30 mg/Kg), GSK3i 71 (10 mg/Kg), or a combination of both were administered by intraperitoneal injection daily for 4 weeks (5-6 mice/treatment group). *p < 0.01 relative to Enzalutamide-treated mice based on pairwise Student t-test.

We observed that inhibition of DNMT1 activity by inhibition of GSK3-induced phosphorylation resulted in re-expression of AR in AR-negative DU145 cells and sensitized these cells to anti-androgens. Downregulation of GSK3 using shRNAs targeting GSK3β (**Supplementary Figure 4A**) or treatment with the DNMT1 inhibitor AzadC resulted in re-expression of the AR protein in DU145 cells (**Figure 5A**). The increase in AR expression resulted in increased expression of prostate specific antigen (PSA), an AR-responsive protein. AR re-expression can be attributed to inhibition of GSK3-mediated DNMT1 phosphorylation (as opposed to other GSK3 targets) because the inhibitory effects of DNMT1 on AR expression was rescued upon expression of DNMT1 S714D (**Figure 5A** and **Supplementary Figure 2A**). GSK3β has been reported to phosphorylate the hinge and ligand binding regions of AR, resulting in decreased expression of AR gene targets (35, 36). Thus, GSK3 inhibition would be expected to not only result in increased AR expression but also in AR activation, and the ensuing increase in expression of AR target genes such as PSA. This could be a basis why PSA expression was observed after GSK3i 71, but not AzadC, treatment. A consequence of AR re-expression is the increase in PSA expression when cells were treated with R1881 concurrent with expression of GSK3 shRNA. The increase in PSA expression was attenuated by treatment with bicalutamide (**Figure 5B**). Utilizing a more selective and potent GSK3 inhibitor, GSK3i 71, we determined that pharmacological inhibition of GSK3, resulted in AR re-expression (**Figure 5C**). Treatment with GSK3i 71 was accompanied by decreased methylated and increased unmethylated of *AR* promoter (**Figure 5D** and **Supplementary Figure 5A**). GSK3i 71-induced AR re-expression were verified at the mRNA levels and using a different AR antibody, which also indicated AR levels in GSK3i 71-treated DU145 cells that were about 18-fold lower than that observed in LNCaP cells (**Supplementary Figures 5B, C**). GSK3 inhibition, but not CDK1 inhibition, also resulted in AR re-expression. Only low AR expression was evident when DNMT1 S714A was expressed, perhaps due to competition of DNMT1 S714A with endogenous DNMT1 WT (**Supplementary Figures 5C**). The increased responsiveness to androgen and antiandrogens upon downregulation of GSK3 expression was also evident in proliferation of DU145 (**Figure 5E**). The combination treatment with Enzalutamide and GSK3i 71 resulted in attenuated proliferation, colony formation, and growth of xenografted DU145 cells when compared to treatment with either agent alone, likely due to expression of AR (**Figures 5F, G** and **Supplementary Figures 5D, E**).

### Restoration of ER Expression in TNBC Cell Lines

**Figure 6:**
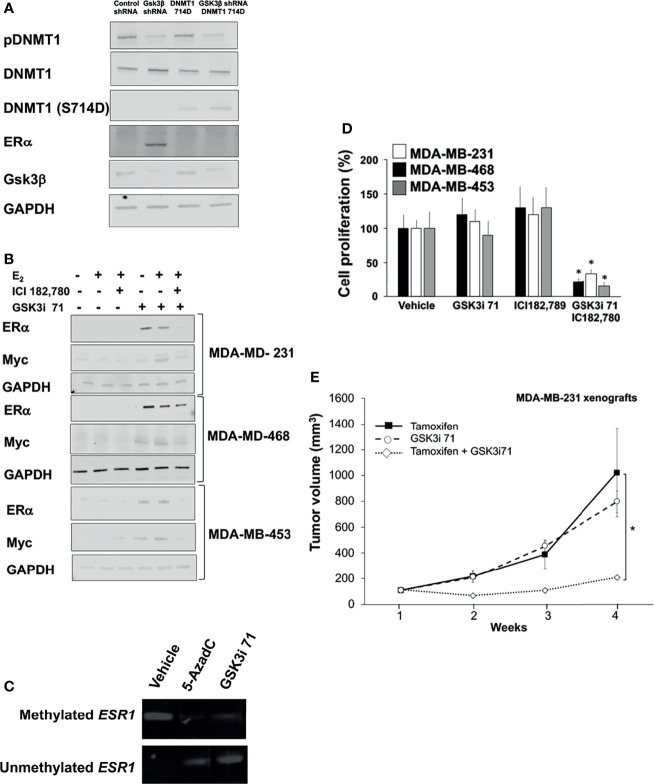
GSK3 inhibition resulted in re-expression of ER and hormone dependence of TNBC lines. **(A)** MDA-MB-231 cells were transfected with expression vector for DNMT1 (S714D) and/or GSK3β shRNA and processed for western blot analyses of indicated proteins normalized to GADPH. Figures are representative of at least 3 independent experiments. **(B)** TNBC lines were treated with 17β-Estradiol (E_2_, 10 nM, 8 h), ICI182,780 (1 µM, 8 h), and/or GSK3i 71 (500 nM, 72 h). Cell were processed for western blot analyses of indicated proteins normalized to GAPDH. Figures are representative of at least 3 independent experiments. **(C)** MDA-MB-231 cells were treated with vehicle, 5-AzadC (5 µM), or GSK3i 71 (500 nM) for 72 h Cells were harvested and genomic DNA extracted, followed by methylation-specific PCR at the promoter region of the *ESR1* gene using bisulfite-treated DNA. Figures are representative of at least 3 independent experiments. **(D)** TNBC lines were treated with ICI182,780 (1 µM), and/or GSK3i 71 (500 nM). After 5 days, WST-1 solution (10 mg/ml) was added to each well. The formazan crystals formed inside the viable cells were measured at 450 nm using a microplate reader. Figures are representative of at least 3 independent experiments *P < 0.01 *vs*. vehicle treated cells. **(E)** Female NSG mice were implanted with MDA-MB-231 cells and treated with tamoxifen (75 mg/Kg), GSK3i 71 (10 mg/Kg), or a combination of both were administered by intraperitoneal injection daily for 4 weeks (5-6 mice/treatment group). *p < 0.01 relative to tamoxifen-treated mice based on pairwise Student t-test.

Epigenetic silencing through promoter hypermethylation of ER accounts for a portion of ER gene silencing, which occurs in 5–49% of patient samples (37, 38). About 30% of invasive BCa are hormone independent because they lack ER expression due to hypermethylation of ER promoter (9, 10). Our data indicates that GSK3β downregulation *via* shRNA or pharmacological inhibition resulted decreased levels of phospho-DNMT1 (S714) and ERa re-expression in TNBC cell lines (**Figures 6A, B** and **Supplementary Figures 4B**). Treatment with GSK3i 71 was accompanied by decreased methylated and increased unmethylated of *ESR1* promoter (**Figure 6C** and **Supplementary Figure 6A**). GSK3i 71-induced ER re-expression was verified at the mRNA level and using a different ER antibody, which also indicated ER levels in GSK3i 71-treated MDA-MB-231 cells that were about 8-fold lower than that observed in MCF7 cells (**Supplementary Figures 6B, C**). GSK3 inhibition, but not CDK1 inhibition, also resulted in ER re-expression. Only low ER expression was evident when DNMT1 S714A was expressed, perhaps due to competition of DNMT1 S714A with endogenous DNMT1 WT (**Supplementary Figures 6C**). ERα re-expression in these lines resulted in 17β-Estradiol (E2)-dependent Myc expression, and the dependence on ER is verified by attenuation of Myc expression in cells treated with ER degrader, ICI182,780 (**Figure 6B**). Other consequence of ERα re-expression is that treatment with GSK3i 71 and ICI182,780 together attenuated cell proliferation and colony formation, but not when either agent was administered alone (**Figures 6D** and **Supplementary Figure 6D**). The combination treatment also resulted in attenuated growth of xenografted MDA-MB-231 cells when compared to treatment with either agent alone, likely due to expression of ERα (**Figure 6E** and **Supplementary Figures 6E, F**). Together these data suggest the restoration of hormone dependence in TNBC cell lines upon downregulation or inhibition of GSK3β.

### Inhibition of DNMT1 Phosphorylation *via* Insulin Like Growth Factor (IGF-1) and Epidermal Growth Factor (EGF)

**Figure 7:**
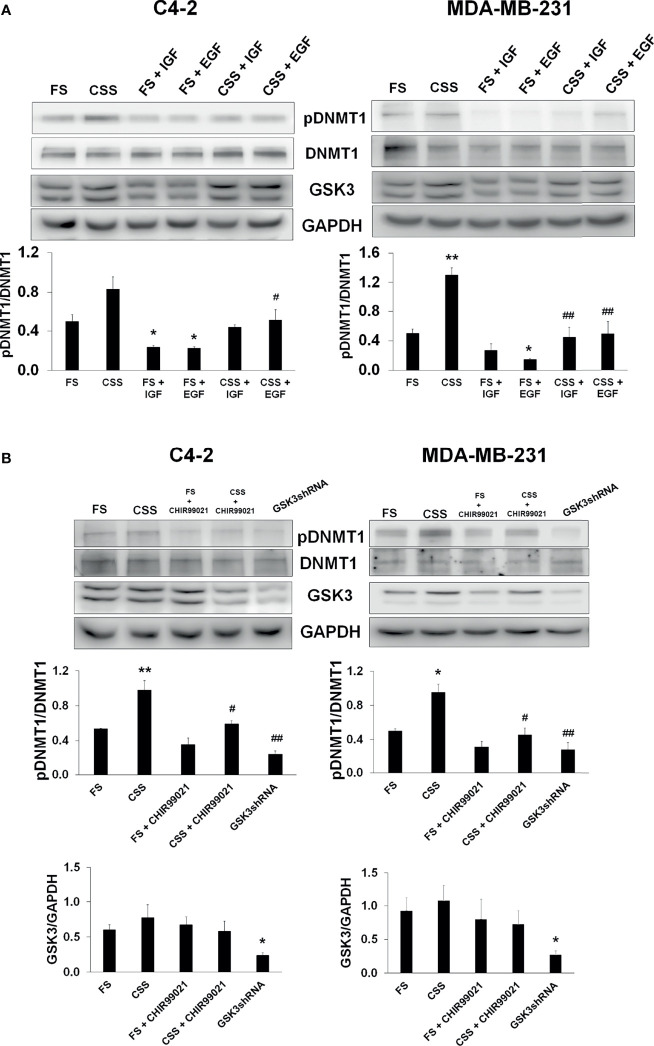
Inhibition of GSK3-mediated DNMT1 phosphorylation by growth factors. **(A)** C4-2 and MDA-MB-231 were grown in media supplemented with Full Serum (FS) or Charcoal Stripped Serum (CSS) and treated with either IGF (1 µM, 4 h) or EGF (1 µM, 4h). Cells were processed for western blot analyses of expression of pDNMT1 and GSK3 normalized to DNMT1 and GAPDH. Figures are representative of at least 3 independent experiments. *P < 0.05 and **P < 0.01 *vs*. FS. ^#^P < 0.05 and ^##^P < 0.01 *vs*. CSS. **(B)** C4-2 and MDA-MB-231 cells were grown in media supplemented with Full Serum (FS) or Charcoal Stripped Serum (CSS) treated with GSK3 inhibitor (CHIR99021) or transfected with GSK3 shRNAs targeting GSK3α and GSK3β. Cells were processed for western blot analyses of indicated proteins normalized to GAPDH. Figures are representative of at least 3 independent experiments. *P < 0.05 and **P < 0.01 *vs*. FS. ^#^P < 0.05 and ^##^P < 0.01 *vs*. CSS.

Since growth factors are key regulators of kinase signaling pathways, including GSK3β, we determined if growth factors regulate the phosphorylation of DNMT1. Growth factor-induced phosphorylation at N-terminal regions of GSK3α and GSKβ and S21 and S9 residues of GSK3α and GSKβ, respectively, have been reported to inhibit GSK3 (39–41). Growth factors would then be expected to inhibit DNMT1 phosphorylation. Along this line, we observed enhanced DNMT1 phosphorylation in C4-2 and MDA-MB-231 cells grown in media supplemented with charcoal-stripped serum (CSS) compared to cells grown in media supplemented with full serum (**Figure 7A**). The inhibitory effects of growth factors on GSK3 activity were verified upon addition of IGF-1 or EGF to CSS-supplemented media and the resulting decrease in the phosphorylation of DNMT1 (**Figure 7A**). Addition of the GSK3 inhibitor, CHIR99021, to CSS-supplemented media also resulted in decreased levels of phosphorylated DNMT1 (**Figure 7B**). Comparable attenuation of DNMT1 phosphorylation was observed upon shRNA-mediated GSK3 inhibition (**Figure 7B**). These experiments support inhibitory effects of growth factors on DNMT1 phosphorylation by the attenuation of GSK3 activity.

## Discussion

In the present study, we report that DNMT1 is a substrate of GSK3, and that phosphorylation on S714 by GSK3 plays a role in modulating DNMT1 activity. The reduction in DNMT1 activity by inhibiting GSK3β-mediated phosphorylation of DNMT1 resulted in the re-expression of hormone receptors AR and ER in PCa and BCa cells, respectively, and re-sensitized these cancers to antihormones.

We observed that IGF-1 and EGF-1 inhibited phosphorylation of DNMT1, perhaps through downregulation of GSK3 activity. IGF-1 can activate Akt, resulting in inactivation of GSK3β (42). GSK3β has been reported to phosphorylate DNMT1 at S410 and S414 and induce proteasomal degradation of DNMT1 (15). However, we did not observe alterations in DNMT1 levels with IGF-1 treatment or upon downregulation of GSK3β *via* shRNA, suggesting cell type specific regulation of DNMT1 levels by GSK3. Breast cancer cells which express human epidermal growth receptor 2 (HER2), which is activated mainly by EGF-1, exhibit different methylation patterns when compared to breast cells that do not express HER2 (43). However, underlying mechanisms were not well-defined. Our studies provide a potential mechanism for this action of EGF-1.

In addition to modulation of DNMT1 stability by phosphorylation (44, 45), we and other have identified DNMT1 post-translational modifications that result in modulation of DNMT1 activity. The first reported DNMT1 phosphorylation was at S515, which regulates the interaction between the regulatory and catalytic domains (46, 47). Phosphorylation of DNMT1 by casein kinase 1delta/epsilon modulates DNA-binding activity DNMT1 (48). Several lysine residues were also reported to be acetylated on DNMT1, including K749, whereas a histone deacetylase SIRT1 physically associates with DNMT1 and deacetylates acetylated DNMT1 (2). Deacetylation of DNMT1 by SIRT1 impairs the ability of DNMT1 to regulate cell cycle G2/M transition. Conversely, inhibition of SIRT1 augments the silencing effects of DNMT1 on the expression of tumor suppressor genes *ESR1* and *CDH1* in MDA-MB-231 cells (49). We identified a DNMT1 phosphorylation site on S714, which is part of the 62-amino acid-long autoinhibitory linker (693-754) that functions to occlude DNA from the active site of DNMT1 to prevent the methylation of unmethylated DNA (17). While previous phosphoproteomic analysis revealed phosphorylation of S714 of DNMT that resulted in altered expression of DNMT1 target genes (32), the basis for the regulation of DNMT1 activity and consequences were not well-defined. We hypothesized that the phosphorylation at S714 might be involved in the catalytic function of DNMT1 due to its close proximity with the cysteine rich CXXC domain, which is reported to be involved in DNMT1 enzymatic activity by sensing hemimethylated DNA and establishing contacts with DNA (18). Our hypothesis was corroborated by Molecular Dynamics simulation that indicates that phosphorylation of S714 stabilized the autoinhibitory linker, inactivated its ability to block the methylation of unmethylated DNA, and increased DNMT1 activity, with the latter being validated using biochemical assays.

In the current study, GSK3 inhibition resulted in decreased DNMT1 activity, re-expression of the AR and ER protein, and re-sensitization to endocrine therapies. PCa is initially androgen dependent but eventually becomes androgen resistant after androgen deprivation therapy. Genetic alterations without loss of AR expression alter the sensitivity of the AR to androgens and are thought to play key roles in the development of androgen-independent advanced PCa. These alterations include AR gene mutation and amplification (50), and induction of bypass pathways in AR signaling, which include intracrine androgen biosynthesis in PCa, expression of constitutively active, ligand-independent AR splice variants, and non-canonical activation of AR by receptor tyrosine kinases (RTK) in the absence of ligand (51). However, 20–30% of advanced metastatic hormone independent PCa is characterized by heterogeneous loss of AR expression resulting from transcriptional silencing that does not involve deletion or mutational mechanisms. Both clinical tumors and PCa cell lines display transcriptional silencing of *AR* due to hypermethylation of its promoter or histone deacetylation (8, 28). Previous studies have shown that more than 25% of ER-negative BCa cells have aberrant methylation status of the *ESR1* promoter (9, 10, 37, 52). Thus, therapeutics aimed at re-expression of AR and ER could impact the targeting of a significant proportion of AR- and ER-negative cancers, respectively.

While *in vitro* and *in vivo* experiments have shown that DNMT1 inhibitor 5-AzaC has antitumor activities in several types of cancer (51–55), there is a need for optimization of treatments targeting DNMT1 activity. Treatment with DNMT inhibitors 5-AzaC and 5,6-dihydro-5-azacytidine could restore sensitivity of androgen insensitive human prostate carcinoma cell lines to growth inhibition by anti-androgens (8, 53, 54). Two nucleoside analogues, 5-azacytidine (5-AzaC, Vidaza) and 5-azadeoxycytidine (5-AzadC, Decitabine), have been approved by Food and Drug Administration (FDA) for the treatment of myelodysplastic syndromes (MDS (55, 56). Although these compounds are effective in the treatment of hematologic conditions, clinical trials with these compounds for the treatment of solid tumors, including PCa, have not shown significant efficacy due to associated side effects (57–59). DNMT1 inhibitors have also not exhibited sufficient efficacy in BCa patients. In a phase I study, 5-AzaC in combination with valproic acid conferred stable disease in one of four advanced BCa patients (60). A phase II clinical trial involving combination of the HDAC inhibitor Entinostat with 5-AzaC did not yield significant clinical benefits, with all 13 TNBC patients not exhibiting partial response (61).

GSK3 is a potential therapeutic target in multiple cancers (62–65). It can act as a tumor promoter as indicated by the elevated levels of GSK3 present in colon cancer (66), pancreatic cancer (67, 68), and leukemia (69, 70). Studies using PCa cells show complex actions of GSK3. The reported tumor suppressor function of GSK3β involves phosphorylation of the hinge and ligand binding regions of AR, resulting in decreased activation of AR gene targets and inhibition of cell proliferation (35, 36). In contrast, both GSK3 isoforms were associated with activation of AR and AR-regulated genes. GSK3β regulates AR localization in the nucleus by phosphorylating AR at S650, and GSK3 inhibitors induced rapid nuclear export of the AR (71). Silencing experiments also indicate that AR transcriptional activity is dependent on GSK3α (72). The diverse actions of GSK3 in cancer cells complicate the development of GSK3 inhibitors as cancer therapeutics. While there is an abundance of data obtained from preclinical models (73), no GSK3 small molecule inhibitor has been approved for cancer therapy. In the current study, we used a more selective and potent inhibitor of GSK3 to downregulate DNMT1 activity, resulting in re-expression of AR and ER. Our study in hormone-dependent cancers supports the potential of novel combinatorial approach involving the administration of GSK3 inhibitors with DNMT inhibitors to augment the sensitivity of PCa and BCa cells to existing inhibitors of the AR and ER, respectively.

## Data Availability Statement

The original contributions presented in the study are included in the article/**Supplementary Material**. Further inquiries can be directed to the corresponding author.

## Ethics Statement

The animal study was reviewed and approved by CWRU IACUC.

## Author Contributions

VS and MM performed study concept and design. VS, JJ, I-JY, YD, and MM provided acquisition, analysis and interpretation of data, and statistical analysis. VS and MM prepared the manuscript in partnership with DB and DW. All authors contributed to the article and approved the submitted version.

## Funding

This work is supported by National Institute of Health grant CA195558 to MM and R43CA243833 to DW.

## Conflict of Interest

MM is co-founder of Oncostatyx. DW is co-founder of CuronBiotech.

The remaining authors declare that the research was conducted in the absence of any commercial or financial relationships that could be construed as a potential conflict of interest.

## Publisher’s Note

All claims expressed in this article are solely those of the authors and do not necessarily represent those of their affiliated organizations, or those of the publisher, the editors and the reviewers. Any product that may be evaluated in this article, or claim that may be made by its manufacturer, is not guaranteed or endorsed by the publisher.
