# Supplementary material for: Re-Expression of ERα and AR in Receptor Negative Endocrine Cancers via GSK3 Inhibition
Source: Front Oncol. 2022 Mar 24;12:824594. doi: 10.3389/fonc.2022.824594 (PMC8988137; doi:10.3389/fonc.2022.824594)
Supplement: Supplementary file 1 [file DataSheet_1.pdf]

## Supplementary figure legends

Supplementary Figure 1. **Expression of Myc-DNMT1 (WT, S714A, and S714D) and GSK3 in transfected cell analyzed for DNMT1 activity**. **(A)** C4-2 and MDA-MB-231 cells were transfected with expression vector for Myc-DNMT1, Myc-DMT1 (S714A), or Myc-DNMT1 (S714D). Cells were processed for western blot analyses of expression of WT and mutant DNMT1 normalized to GAPDH. **(B)** C4-2 and MDA-MB-231 cells were transduced with control shRNA or GSK3 shRNA lentiviruses targeting GSK3α and GSK3β and selected using puromycin. Cells were processed for western blot analyses of expression of GSK3 normalized to GAPDH. Blots are representative of three independent experiments.

Supplementary Figure 2. **Expression of Myc-DNMT1 (WT, S714A, and S714D) and GSK3 in transfected cells processed for ChIP (A)** DU145 and MDA-MB-231 cells were transfected with expression vectors: Myc-DNMT1, Myc-DNMT1 (S714A), or Myc-DNMT1 (S714D). Cells were processed for western blot analyses of expression of WT and mutant DNMT1 normalized to GAPDH. **(B)** DU145 and MDA-MB-231 cells were transduced with control or GSK3 shRNA lentiviruses targeting GSK3α and GSK3β followed by puromycin selection. Cells were processed for western blot analyses of expression of GSK3 normalized to GAPDH. Figures are representative of at least 3 independent experiments. **(C)** DU145 and MDA-MB-231 cells were treated with either CHIR99021 (4 μM) or RO-3306 (1 μM) for 4 h. Cells were then processed for ChIP analyses of recruitment of DNMT1 to the *AR* promoter in DU145 cells and to the first exon of the *ESR1* gene in MDA-MB-231 cells. Input DNA was used as normalization control. IgG control was used as a negative control. Figures are representative of at least 3 independent experiments.

Supplementary Figure 3. **Ribbon representation of phosphorylated and unphosphorylated protein-DNA complex. A, C** & **D** represent only the region of superimposed phosphorylated and unphosphorylated 100 amino acids long autoinhibitory loop at different viewing angles, while the other part of the protein is hidden. **(B)** represents the entire phosphorylated and unphosphorylated protein-DNA complex.

Supplementary Figure 4. **Expression of Myc-DNMT1 S714D and GSK3 in transfected cells. (A)** DU145 cells or **(B)** MDA-MB-231 cells were transduced with control shRNA- or GSK3β shRNA-lentiviruses (2 different clones) followed by puromycin selection. Cells were processed for western blot

analyses of expression of GSK3β, AR, and/or ERα, normalized to GAPDH. Blots are representative of three independent experiments.

Supplementary Figure 5. **Expression of AR in DU145 cells and xenografts and colony formation upon treatment with GSK3i 71.** DU145 cells were treated as indicated followed by **(A)** extraction of genomic DNA and methylation-specific PCR of the promoter region of the *AR* gene using bisulfite-treated DNA and **(B)** qRT-PCR analyses of *AR* expression **(C)** Western blot analyses of AR expression using whole cell extracts from DU145 cells treated for 72 h with GSK3i 71 (500 nM), CHIR99021 (4 μM), RO-3306 (1 μM) or transfected with expression vector for Myc-DNMT1 (S714A). Extracts from LNCaP cells were used as a positive control. For (A) and (B) *$P < 0.05$ vs. Control. Figures are representative of at least 3 independent experiments. **(D)** Immunohistochemical detection of AR in DU145 xenografts obtained from male NSG mice treated with Enzalutmide (30 mg/Kg) ± GSK3i 71 (10 mg/Kg). Panels represent images from 5 pairs of mice. Scale bars are 100 mm. **(E)** DU145 colony formation upon treatment with vehicle, Enzalutamide (10 mM), GSK3i 71 (100 nM), or Enzalutamide + GSK3i 71

Supplementary Figure 6. **Expression of ERα in MDA-MB-231 cells and xenografts and colony formation upon treatment with GSK3i 71.** MDA-MB-231 cells were treated as indicated followed by **(A)** extraction of genomic DNA and methylation-specific PCR of the promoter region of the *AR* gene using bisulfite-treated DNA and **(B)** qRT-PCR analyses of *ESR1* expression **(C)** Western blot analyses of ERα expression using whole cell extracts from MDA-MB-123 cells treated for 72 h with GSK3i 71 (500 nM), CHIR99021 (4 μM), RO-3306 (1 μM) or transfected with expression vector for Myc-DNMT1 (S714A). Extracts from MCF7 cells were used as a positive control. For (A) and (B) *$P < 0.05$ vs. Control. Figures are representative of at least 3 independent experiments. **(D)** MDA-MB-231 cell colony formation upon treatment with vehicle, ICI182,780 (1 mM), GSK3i 71 (100 nM), or ICI182,780 + GSK3i 71. **(E)** Female NSG mice bearing MDA-MB-231 xenografts and treated with tamoxifen (75 mg/Kg) ± GSK3i 71 (10 mg/Kg). Shown are Western blot analyses of ERα. Blots were probed for GAPDH as a loading control. **(F)** Immunohistochemical detection of ERα in MDA-MB-231 xenografts. Scale bars are 100 μm.

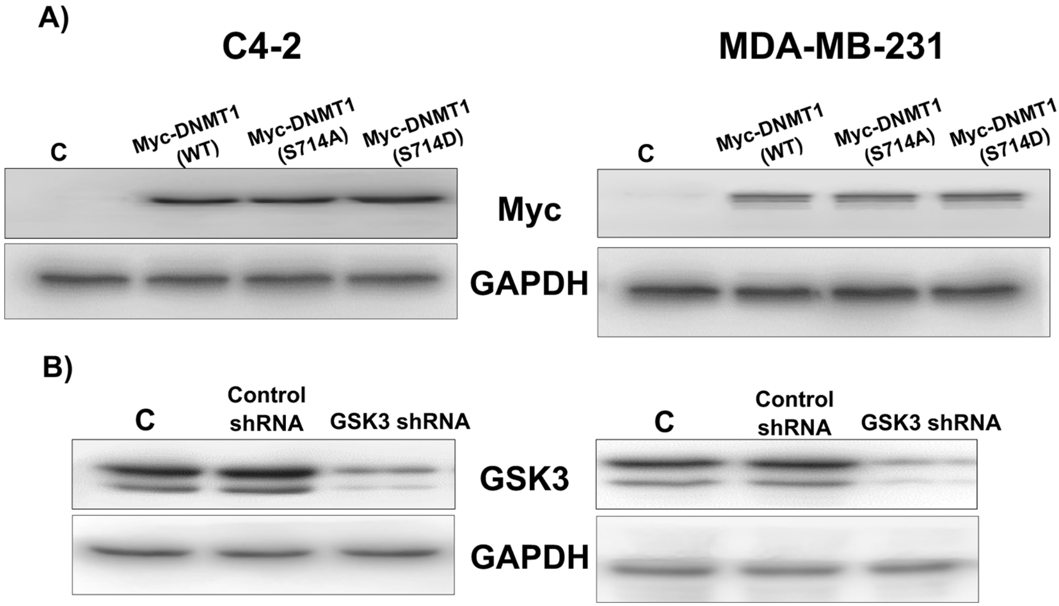
A)
C4-2
MDA-MB-231
C
Myc-DNMT1 (WT)
Myc-DNMT1 (S714A)
Myc-DNMT1 (S714D)
Myc
GAPDH
B)
C
Control shRNA
GSK3 shRNA
GSK3
GAPDH

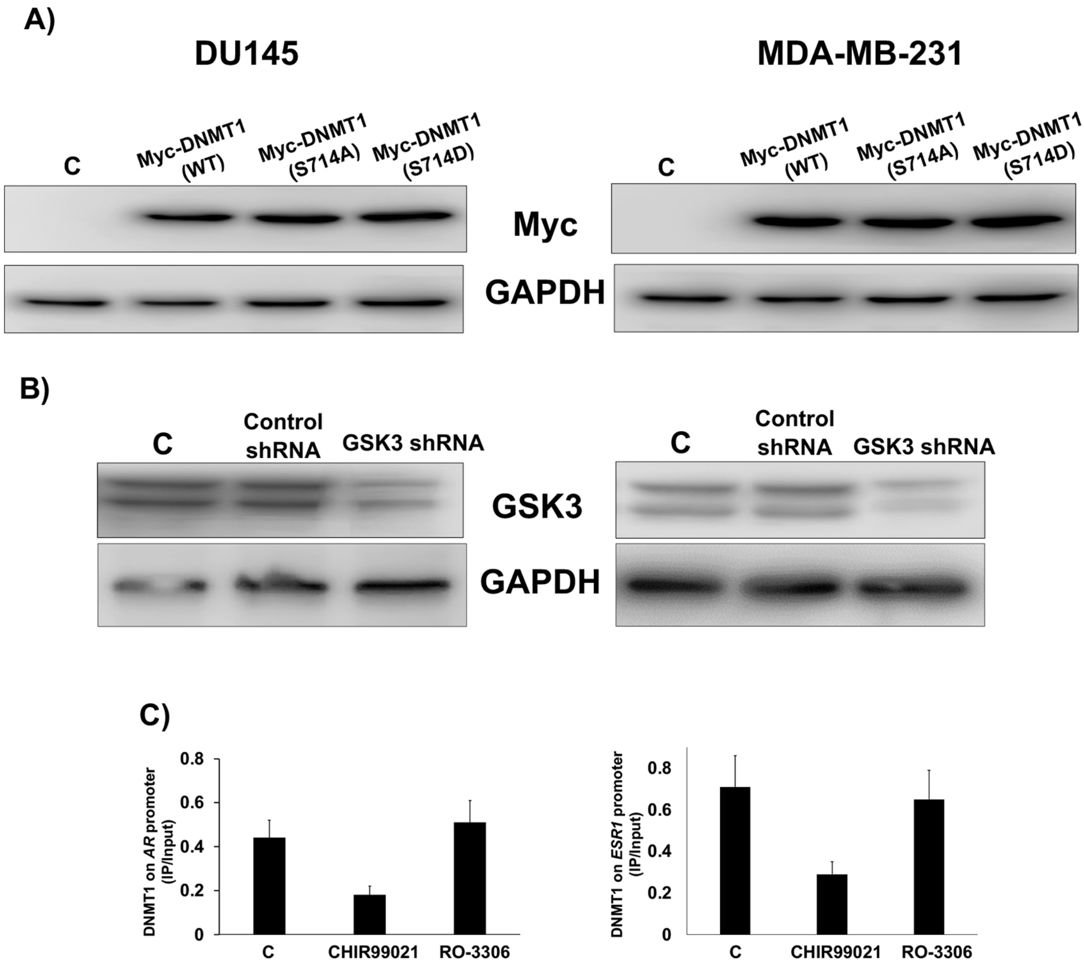

A)
DU145
MDA-MB-231
C
Myc-DNMT1 (WT)
Myc-DNMT1 (S714A)
Myc-DNMT1 (S714D)
Myc
GAPDH
B)
C
Control shRNA
GSK3 shRNA
GSK3
GAPDH
C)
DNMT1 on AR promoter (IP/Input)
0
0.2
0.4
0.6
0.8
C
CHIR99021
RO-3306
DNMT1 on ESR1 promoter (IP/Input)

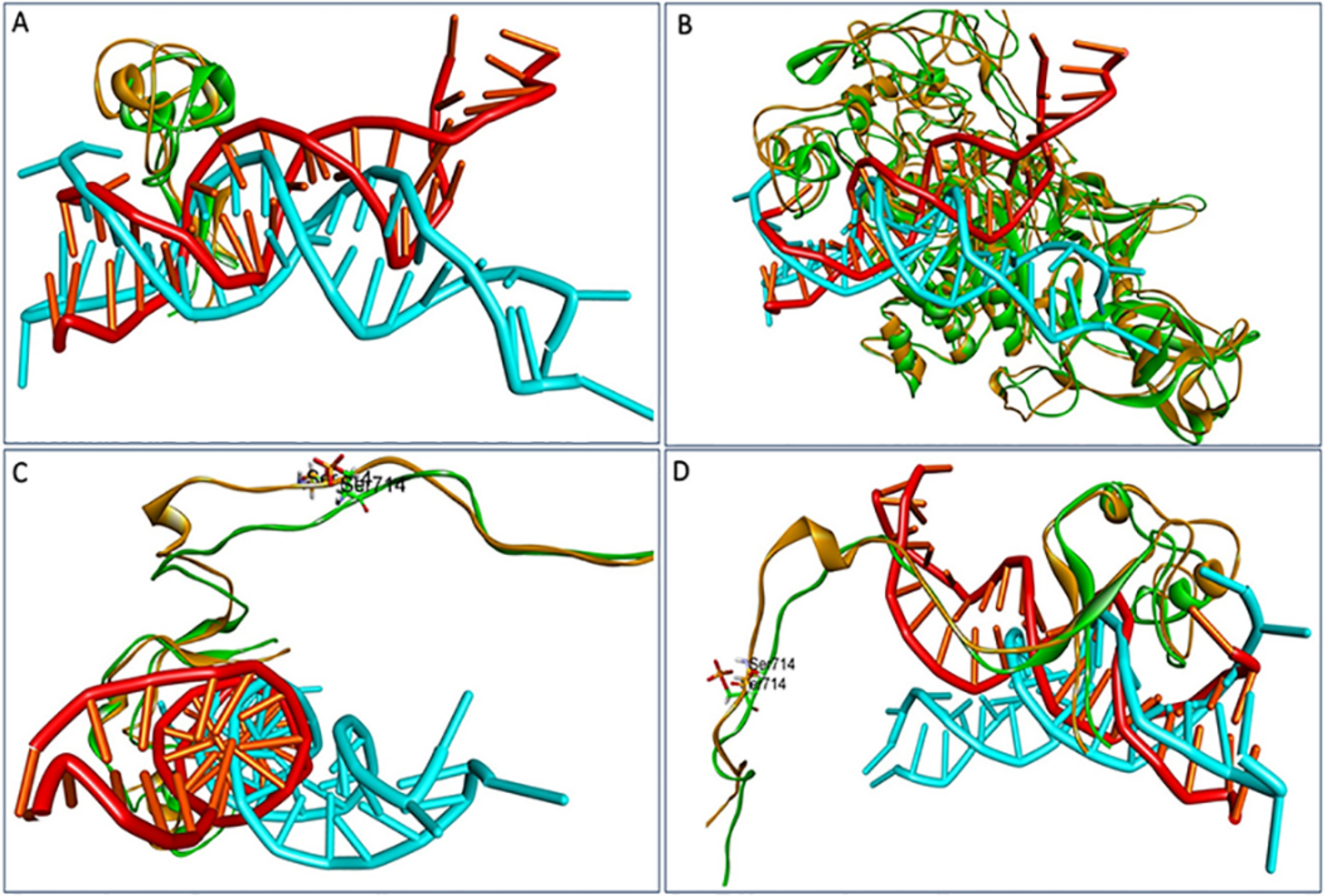
A
B
C
Ser714
D
Ser714

A)

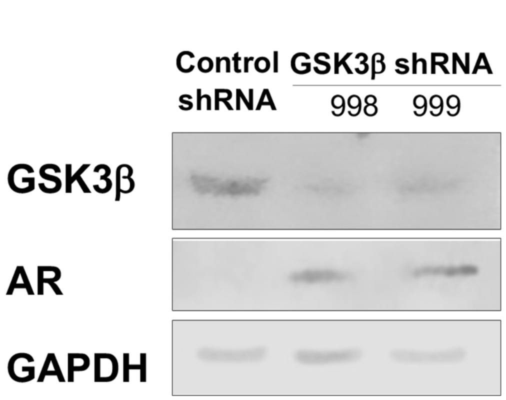
DU145
Control shRNA
GSK3β shRNA
998
999
GSK3β
AR
GAPDH

B)

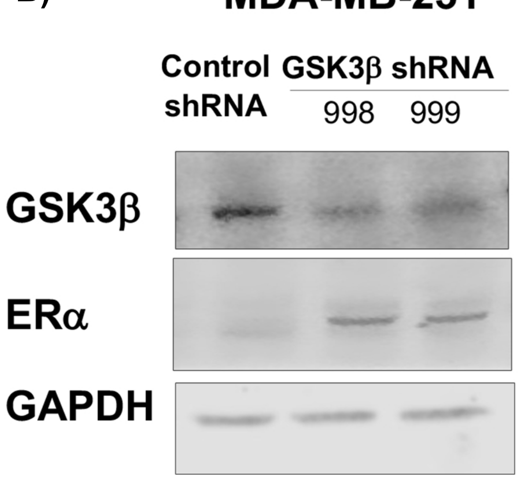
MDA-MB-231
Control shRNA
GSK3β shRNA
998
999
GSK3β
ERα
GAPDH

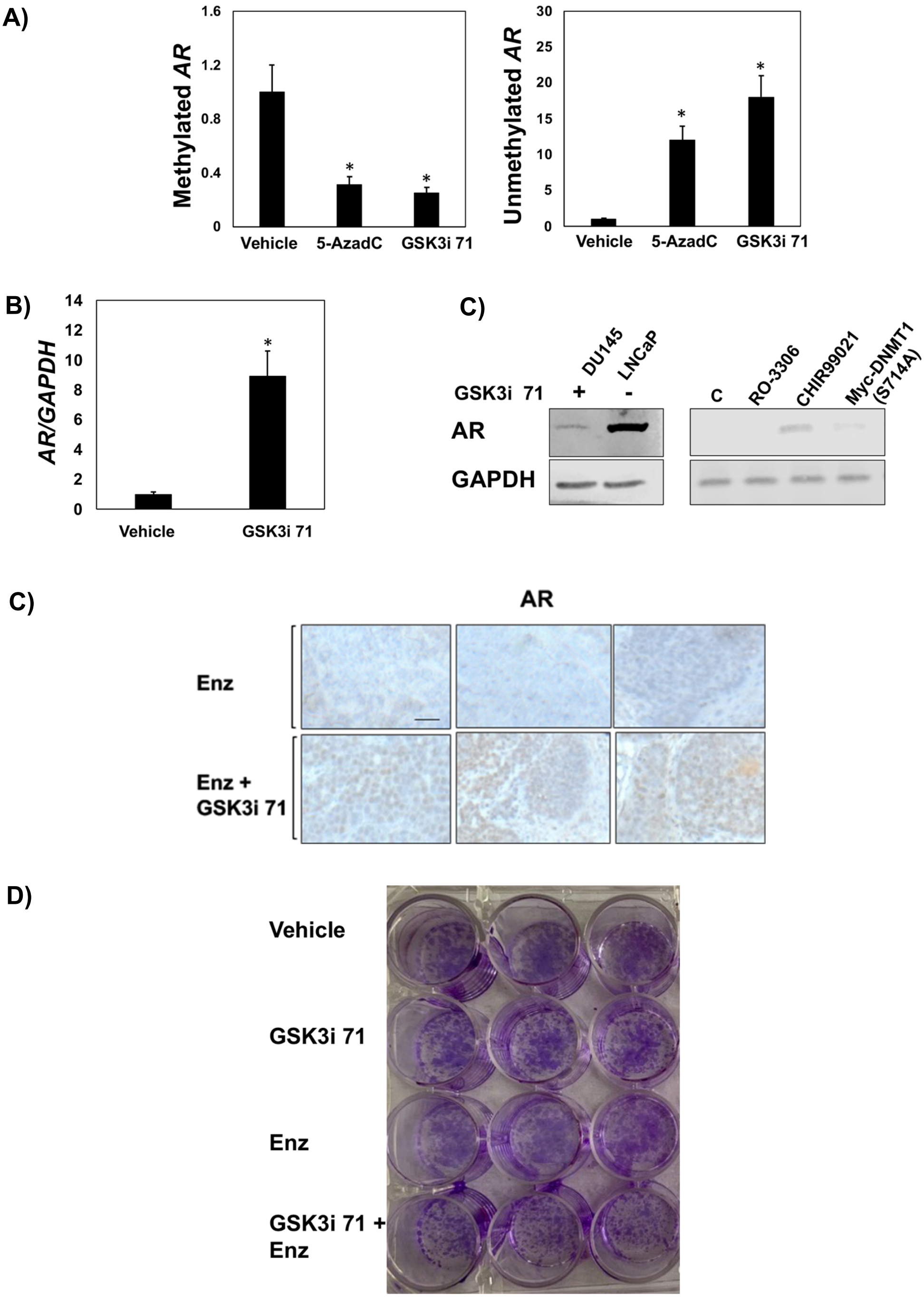
A)
Methylated AR
1.6
1.2
0.8
0.4
0
*
*
Vehicle
5-AzadC
GSK3i 71
Unmethylated AR
30
25
20
15
10
5
0
*
*
Vehicle
5-AzadC
GSK3i 71
B)
AR/GAPDH
14
12
10
8
6
4
2
0
*
Vehicle
GSK3i 71
C)
DU145
LNCaP
GSK3i 71
+
-
C
RO-3306
CHIR99021
Myc-DNMT1 (S714A)
AR
GAPDH
C)
AR
Enz
Enz + GSK3i 71
D)
Vehicle
GSK3i 71
Enz
GSK3i 71 + Enz

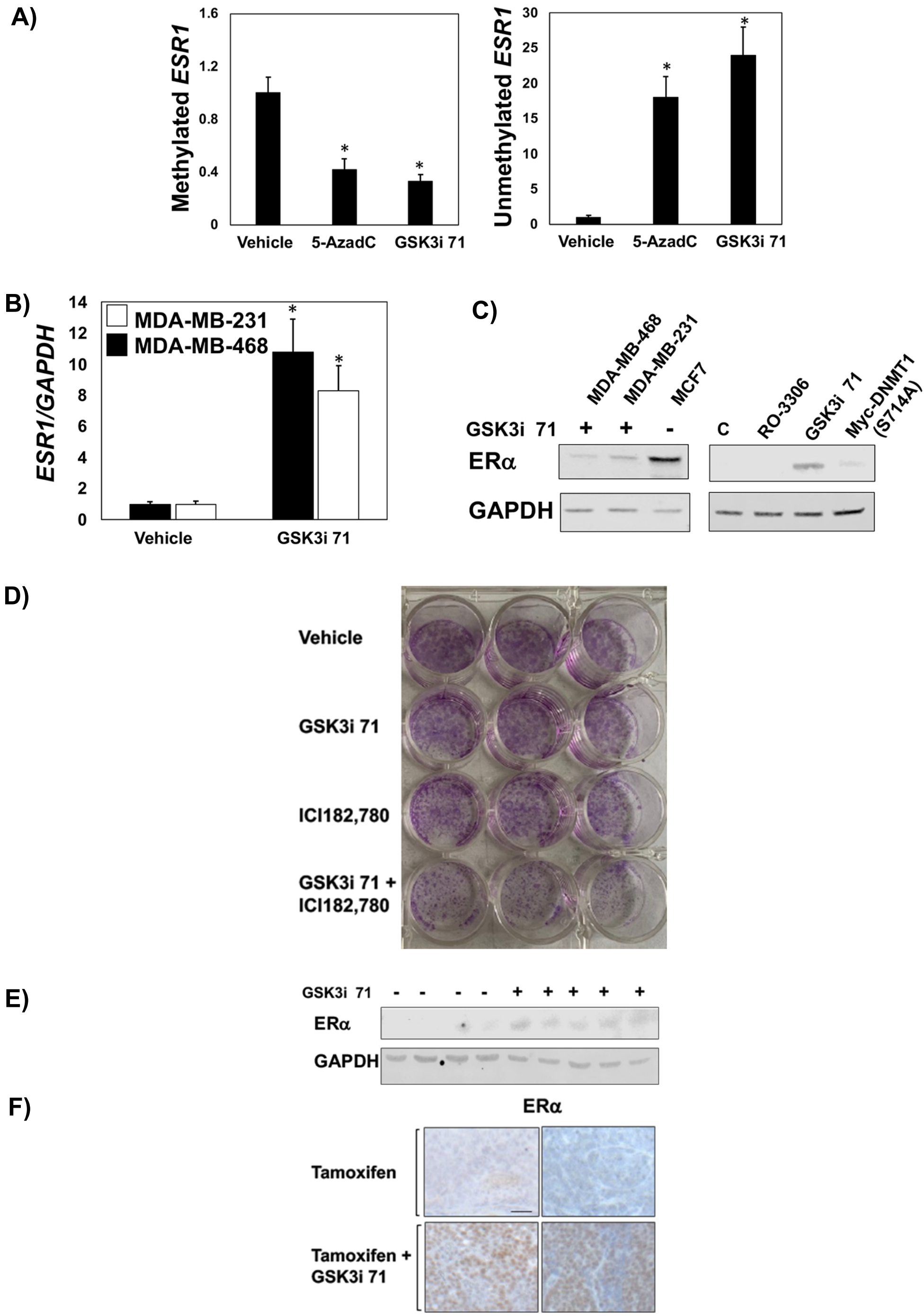
A)
Methylated ESR1
1.6
1.2
0.8
0.4
0
*
*
Vehicle
5-AzadC
GSK3i 71
Unmethylated ESR1
30
25
20
15
10
5
0
*
*
Vehicle
5-AzadC
GSK3i 71
B)
ESR1/GAPDH
14
12
10
8
6
4
2
0
MDA-MB-231
MDA-MB-468
*
*
Vehicle
GSK3i 71
C)
MDA-MB-468
MDA-MB-231
MCF7
GSK3i 71
+
+
-
C
RO-3306
GSK3i 71
Myc-DNMT1 (S714A)
ERα
GAPDH
D)
Vehicle
GSK3i 71
ICI182,780
GSK3i 71 + ICI182,780
E)
GSK3i 71
- - - - + + + + +
ERα
GAPDH
F)
ERα
Tamoxifen
Tamoxifen + GSK3i 71
